# Determination and Application of Nineteen Monoamines in the Gut Microbiota Targeting Phenylalanine, Tryptophan, and Glutamic Acid Metabolic Pathways

**DOI:** 10.3390/molecules26051377

**Published:** 2021-03-04

**Authors:** Shu-Rong Ma, Jin-Bo Yu, Jie Fu, Li-Bin Pan, Hang Yu, Pei Han, Zheng-Wei Zhang, Ran Peng, Hui Xu, Yan Wang

**Affiliations:** State Key Laboratory of Bioactive Substance and Function of Natural Medicines, Institute of Materia Medica, Chinese Academy of Medical Sciences/Peking Union Medical College, Beijing 100050, China; mashurong@imm.ac.cn (S.-R.M.); yujinboyjb@163.com (J.-B.Y.); fujie@imm.ac.cn (J.F.); panlibin@imm.ac.cn (L.-B.P.); yuhang@imm.ac.cn (H.Y.); hanpei@imm.ac.cn (P.H.); zhangzhengwei@imm.ac.cn (Z.-W.Z.); pengran@imm.ac.cn (R.P.); xuhui@imm.ac.cn (H.X.)

**Keywords:** monoamine neurotransmitters, liquid chromatography–tandem mass spectrometry (LC-MS/MS), nervous system (NVS), gut microbiota, tryptophan, phenylalanine, glutamic acid

## Abstract

It has been reported that monoamine neurotransmitters can be produced by gut microbiota, and that several related metabolites of amino acids in these pathways are associated with nervous system (NVS) diseases. Herein, we focused on three pathways, namely, phenylalanine (Phe), tryptophan (Trp), and glutamic acid (Glu), and established an underivatized liquid chromatography–tandem mass spectrometry (LC-MS/MS) method for the quantification of nineteen monoamine neurotransmitters and related metabolites in the gut microbiota. The neurotransmitters and related metabolites included Phe, tyrosine (Tyr), l-dopa (Dopa), dopamine (DA), 3-methoxytyramine, Trp, hydroxytryptophan, 5-hydroxytryptamine (5-HT), 5-hydroxyindole-3-acetic acid (5-HIAA), kynurenine (KN), kynurenic acid (KYNA), melatonin, tryptamine (TA), indole-3-lactic acid (ILA), indole-3-acetic acid (IAA), indolyl-3-propionic acid (IPA), Glu, gamma-aminobutyric acid (GABA), and acetylcholine (Ach). A fluoro-phenyl bonded column was used for separation, and the mobile phase consisted of methanol:acetonitrile (1:1) and water, with 0.2% formic acid in both phases. The compounds exhibited symmetric peak shapes and sufficient sensitivity under a total analysis time of 8.5 min. The method was fully validated with acceptable linearity, accuracy, precision, matrix effect, extraction recovery, and stability. The results showed that neurotransmitters, such as Dopa, DA, 5-HT, GABA, and Ach, were present in the gut microbiota. The metabolic pathway of Trp was disordered under depression, with lower levels of 5-HT, 5-HIAA, KN, KYNA, TA, ILA, IAA, IPA, and Glu, and a higher ratio of KYNA/KN. In addition, some first-line NVS drugs, such as sertraline, imipramine, and chlorpromazine, showed regulatory potential on these pathways in the gut microbiota.

## 1. Introduction

Nervous system (NVS) diseases are causing increasing problems globally [1]. According to the reports of the World Health Organization, fast-paced work or increased pressure cause depression, anxiety, schizophrenia, etc.; while the neurodegenerative diseases caused by aging (such as Alzheimer’s disease and Parkinson’s disease) have influenced the quality of daily life to a considerable extent [2]. The development of disease in NVS is always accompanied by an abnormal transportation and metabolism of neurotransmitters at specific locations in the brain [3]. However, the mechanisms of these diseases are complex and associated with multiple aspects, including genetic, environmental, and physiological factors [4,5]. The mechanisms of NVS diseases have not been fully clarified [6,7].

In recent years, the concept of the “gut–brain” axis has provided new insight into the pathogenic mechanism of NVS diseases. The metabolism of the gut microbes is closely related to emotion and behavior [8], and a chemical dialog connects the host and gut microbiome [3,8]. First, several monoamine neurotransmitters, such as gamma-aminobutyric acid (GABA) [9], tryptamine (TA) [10], catecholamines, and serotonin (5-HT) [11], have been reported to be generated or metabolized by gut microbes. These absorbed molecules in vivo could regulate central and peripheral NVS functions via signaling pathways [12]. Second, gut microbes themselves, or their monoamine metabolites on amino acid catabolism pathways (kynurenine, etc.) can activate neural pathways or the immune system and then affect the neuronal transcription in the host’s brain, leading to the changes in host behavior [13,14]. On the other hand, the central NVS can regulate the status of the gastrointestinal tract or enteric NVS by secreting signal molecules, mediated by sympathetic and parasympathetic branches or the hypothalamus–pituitary–adrenal axis, which correspondingly affect the colonization or the metabolism of intestinal bacteria [15]. Though much remains to be explored, these metabolites on amino acid pathways have been uncovered as signals in the gut microbiota, and they play essential roles in the microbiota–host crosstalk [16].

The reported main amino acid pathways in the NVS and gut microbiota included three pathways: the phenylalanine (Phe) pathway, glutamic acid (Glu) pathway, and tryptophan (Trp) pathway [8,16]. In the Phe pathway in vivo, ingested Phe could be transformed into tyrosine (Tyr) through hydroxylation, and then l-dopa (Dopa) and dopamine (DA) were produced, which were further metabolized into 3-methoxytyramine (MT) [17]. MT is a novel neuromodulator involved in the control of movement [17,18]. Several neurotransmitters could be generated or transformed by gut microbes [19,20]. In the metabolic pathway of Glu, Glu and its metabolite GABA are known as key excitatory or inhibitory neurotransmitters in the NVS, and dysfunctions in signal transduction are closely related to depression [9,21]. Acetylcholine (Ach), an important compound for transmitting information between neurons, participated in the regulation of learning and memory [22], and has been reported to be produced by *Lactobacillus* [23]. The metabolism of Trp has been illustrated in several research studies [8,16,24], and multiple pathways are involved. (1) 5-Hydroxytryptophan (5-HTP) and 5-hydroxytryptamine (5-HT), derived from Trp by a hydroxylase, are related to anxiety and depression, and the subsequent metabolite 5-hydroxyindole-3-acetic acid (5-HIAA) is associated with aggressive and depressive behaviors [25,26]. Another metabolite, melatonin (MLT), is involved in the regulation of sleep [27]. (2) Kynurenine (KN) and kynurenic acid (KYNA) can be generated by bacterial indoleamine 2,3-dioxygenase (IDO) 1 [16], and both of them are regulators in central NVS and gastrointestinal functions [28,29,30]. (3) Tryptamine (TA), produced by the bacterial decarboxylase from Trp, is responsible for mental and physical stress [31], and its derivatives generated in gut microbiota, such as indole-3-acetic acid (IAA), indole-3-propionic acid (IPA), and indole-3-lactic acid (ILA), are signal molecules that regulate intestinal permeability and host immunity, thereby affecting the function of the NVS [16,24]. The roles of these above mentioned monoamine neurotransmitters and metabolites in the pathogenesis of NVS disease (mediated by gut microbiota) are being increasingly unveiled [8,16,20,21,23,24]. Deciphering the internal associations between these pathways might facilitate pathological research and drug development targeting gut microbiota. While a rapid and accurate method, or platform, for the quantification of these molecules is essential.

For years, fluorescence detection (EC) and electrochemical detection (ECD) have been common methods for analyzing monoamine neurotransmitters in body fluids, since they allow quick and inexpensive detection [32,33]. However, their main limitations are that only a subset of molecules can be detected within one sample, and the sensitivity of different neurotransmitters varies greatly [34]. With the development of liquid chromatography–tandem mass spectrometry (LC-MS/MS), several methods have been published targeting neurotransmitters in brain or extracellular fluid [34,35,36,37,38]. However, publications on the level of monoamines in gut microbiota have been sporadic. To our knowledge, no comprehensive method has been reported to quantify these functional monoamines, validated in fecal or intestinal bacterial samples, and for targeting all three pathways on phenylalanine, tryptophan, and glutamic acid. Here, we aimed to establish a simple and sensitive LC–MS/MS method for these nineteen monoamine neurotransmitters and related functional metabolites in the gut microbiota, and aimed to apply it to NVS research and drug screening.

## 2. Results and Discussion

### 2.1. Method Development

**Figure 1:**
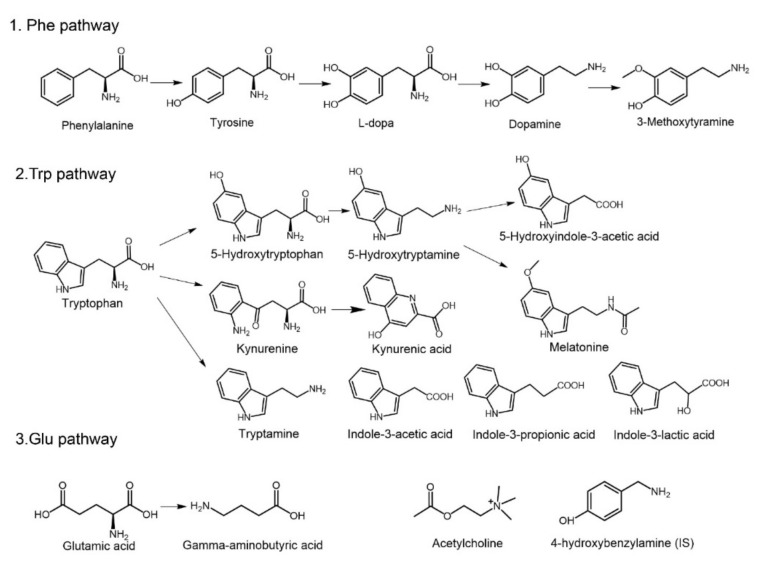
Structures and pathways of the nineteen monoamine neurotransmitters and related metabolites. Phenylalanine (Phe), tyrosine (Tyr), l-dopa (Dopa), dopamine (DA), 3-methoxytyramine (MT), tryptophan (Trp), hydroxytryptophan (5-HTP), 5-hydroxytryptamine (5-HT), 5-hydroxyindole-3-acetic acid (5-HIAA), kynurenine (KN), kynurenic acid (KYNA), melatonin (MLT), tryptamine (TA), indole-3-lactic acid (ILA), indole-3-acetic acid (IAA), indolyl-3-propionic acid (IPA), glutamic acid (Glu), gamma-aminobutyric acid (GABA), acetylcholine (Ach), and 4-hydroxybenzylamine (internal standard, IS).

The development and optimization of this method for the nineteen monoamine neurotransmitters and related metabolites (in Figure 1) included three parts: (1) chromatographic conditions, (2) mass spectrometric conditions, and (3) sample preparation. In this study, considering that most of the metabolites were highly polarized, they could hardly be retained on conventional C_18_ columns, and matrix interference may have occurred. Therefore, a HSS PFP column was used to achieve the separation, the stationary phase of which was high-strength bonded silica with pentafluorophenyl ligand. The separation principle was composed of multiple factors, including hydrogen bonds, dipole–dipole interactions, and aromatic (π−π) and hydrophobic interactions. Then, polar metabolites with similar structures could be successfully separated without interference between peaks, eliminating the complex steps of derivatization treatment. Moreover, this column is favorable for nonprofessional researchers to operate when compared with hydrophilic interaction liquid chromatography columns, which have higher durability like frequently used C_18_ columns. The column temperature was maintained at 35 °C, as suggested.

In terms of the selection of mobile phase, because almost all the neurotransmitters contained amino and hydroxyl groups, the mass spectrometric response of these compounds would increase under acidic conditions, and therefore, the pH value of the mobile phase was adjusted to acidity. Finally, a 0.2% concentration of formic acid was chosen because of its preferable peak shape and acceptable response by comparing various levels of formic acid, acetic acid (0.05%, 0.1%, 0.2%, 0.5%), or the combination of an acid with ammonium formate/ammonium acetate (1 mM, 2 mM, 5 mM, 10 mM). In addition, the combination of methanol and acetonitrile (*v:v* = 1:1) was selected for better response and proper retention time.

The mass spectrometric conditions, including gas flow, gas pressure, and interface temperature, were adjusted according to the response of the metabolites. The collision energy (CE) of detection for each metabolite was optimized separately (as shown in Table 1). In the pretreatment process, the peak shape and stability of the compounds were taken into consideration. Protein precipitation by acetonitrile showed better performance than that by methanol, while some metabolites, such as 5-HT, 5-HIAA, and IAA etc., were unstable upon analysis. Formic acid or ascorbic acid has been reported to increase the stability of neurotransmitters in brain samples [39,40]. However, addition of ascorbic acid led to an increase in the response of 5-HIAA and 5-HT over time, and lowered the response of 5-HTP in the matrix of the gut microbiota. Finally, 2% formic acid was added (to acetonitrile) to stabilize these metabolites before analysis. The drying and resolvation procedures were constructed to obtain a good peak shape and preferable sensitivity.

### 2.2. Method Validation

Currently, there is no unified guideline for the methodical validation of endogenous substances, although this is indispensable in biological analysis. Some strategies have been reported, including (1) adding authentic analytes into the real matrix, (2) adding surrogate analytes via stable isotope-labeled internal standards into the real matrix, and (3) using a surrogate matrix by adding authentic analytes [41]. The second strategy is accepted as the most accurate and convenient strategy for analyzing endogenous substances, while it is relatively expensive and difficult to acquire all nineteen stable isotope-labeled compounds. Preliminary experiments showed that these endogenous analytes could not be completely removed by conventional procedures, including the adsorption of activated carbon or hydrolysis under alkaline conditions. Furthermore, any surrogate matrix, whether water, buffer, synthetic matrix, or treated matrix, may have different matrix effects compared with the untreated authentic matrix, leading to a possible system error in the true level of these compounds in practical samples. Herein, authentic analytes were added to the real matrix for method validation.

#### 2.2.1. Specificity and Carryover

**Figure 2:**
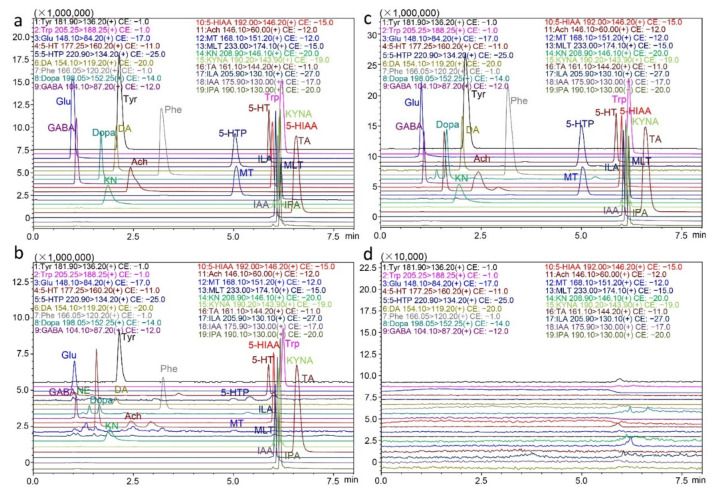
Representative extracted ion chromatograms of the nineteen neurotransmitters and metabolites. (**a**) In standards, (**b**) in practical fecal samples, (**c**) in fecal samples spiked with standards, and (**d**) in blank solvent injected after consecutive injections of high concentrations of analytes.

The specificity of this method is shown using graphs of standards in solvent, authentic samples, and standard-spiked authentic samples (Figure 2a–c). Specificity was evaluated by adding standards to the matrix, and comparing the retention time of the peak that correspondingly increased in area. The retention time of each analyte in the intestinal bacterial sample was identical after addition of standards, and no endogenous interference was observed in the real matrix under current conditions (Figure 2a–c). The carryover effect results showed that after consecutive injections of high concentrations of analytes, no residual effects existed, as shown in Figure 2d.

#### 2.2.2. Linearity and LLOQ

The linearity of the calibration curve was calculated by the least square method in mixed intestinal bacterial samples, with a weighting factor of 1/X for fitting. The retention time (Rt), linear range and correlation coefficient are listed in Table 2. The correlation coefficients of all the analytes were above 0.99, and the nonzero calibrators of every analyte met the acceptance criteria in each validation run, indicating satisfactory linearity over the whole range. Specifically, considering that the levels of those metabolites varied greatly in the intestinal bacterial samples, the linear range of the compounds was established according to their baseline levels. For example, amino acids such as Glu, Tyr, Phe, and Trp were present at micrograms per milliliter in the matrix, and their linear range was correspondingly higher (500–50,000 ng/mL) after adjustment. The lowest limit of quantification (LLOQ) of this method was the lowest point in the calibration curve, ranging from 0.01 ng/mL to 500 ng/mL (with acceptable accuracy and precision). The instrumental lowest limit of detection (LLOD) and LLOQ data were collected in a blank solvent, due to the lack of analyte-free matrix. Results are shown in Table 2 (instrumental LLOD for each analyte, 0.005–5 ng/mL; instrumental LLOQ for each analyte, 0.01–10 ng/mL).

#### 2.2.3. Accuracy and Precision

The intra- and inter-day accuracy and precision assays were performed on three consecutive days with five repeats. The results of each analyte are summarized in Table 3. The intra-day accuracy results (relative error, RE) of the nineteen compounds were within −11.58%–11.49% of the nominal level, and the relative standard derivation (RSD) was in the range of 1.56%–10.15%. The inter-day accuracy results (RE) were within −11.27%–6.69% of the nominal value, and the RSD values were in the range of 2.16%–10.42%. The accuracy and precision results met the quantitative requirements.

#### 2.2.4. Matrix Effect and Extraction Recovery

Matrix effect and recovery assays are important components of method validation in biological analysis, while in terms of endogenous metabolites, the treatment procedures and calculation method are different. Three concentrations of quality control (QC) were used for the matrix effect and recovery studies. The ratio of the increased area of analytes with matrix versus the peak area without matrix was regarded as the matrix effect. The results showed that the matrix effect was consistent at different levels and in the range of 68.24–117.71%, as shown in Table 3. The ratio of the peak area of the QC samples versus the standards directly added to the post-treated matrix was regarded as extraction recovery, and the recovery was in the range of 83.14–117.21% (in Table 3).

#### 2.2.5. Stability

The stability assays were evaluated in three aspects: the short-term stability of samples at 4 °C for 12 h after being added to acetonitrile, the short-term stability of samples at 4 °C for 12 h after sample preparation, and the relatively long-term stability of samples at −70 °C for 2 weeks before sample preparation. The results indicated that all the analytes were stable under these conditions within a range of 85–115% of the nominal value (in Table 3). Repeated freezing–thawing of samples should be avoided for the reason of changes in analyte content.

### 2.3. Neurotransmitters of Rats with Depression in the Gut Microbiota

**Figure 3:**
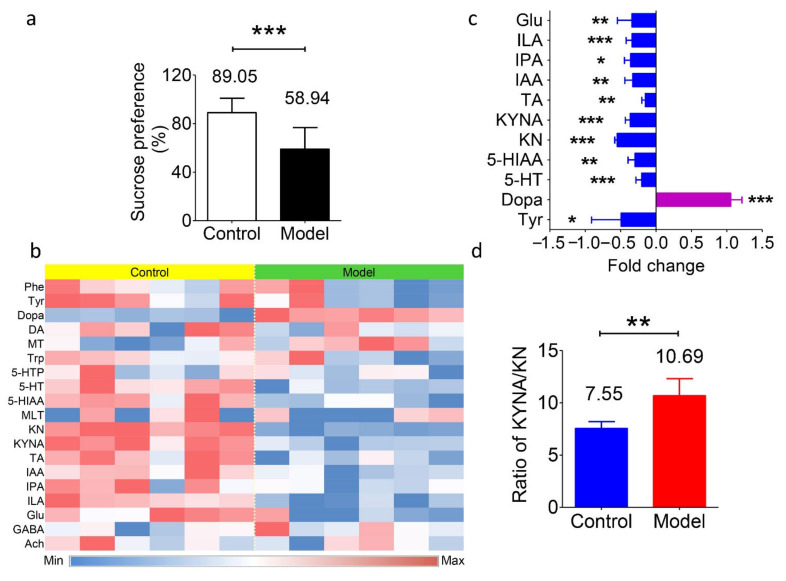
Levels of transmitters and metabolites in fecal samples of the depression model and normal control Sprague-Dawley rats. (**a**) Percentage of sucrose preference in the normal control group (89.05%) and depression model group (58.94%). *** *p* < 0.001. (**b**) Heatmap of the nineteen transmitters and metabolites in fecal samples from both groups. Blue represents a lower level, and red represents a higher level. (**c**) The fold change of the substance levels in the model group versus the control group with significance. * *p* < 0.05, ** *p* < 0.01, *** *p* < 0.001. (**d**) Value of the KYNA/KN ratio in the model group (10.69) and the control group (7.55). * *p* < 0.01. Data in a, c, and d are shown as the means ± SD and were analyzed by two-tailed Student’s *t* test.

The newly established method was used to investigate differences under mental illnesses. Then, a rat model of chronic unpredictable stress depression was established. After an eight-week unpredictable stimulation, the depression model was successfully established. Then, fresh fecal samples from both the model and control groups were collected for analysis. The variations in behavior were monitored and recorded by the sucrose preference test. Rats with depression showed a significantly lower trend to drink sucrose water (in Figure 3a), suggesting anhedonia in the rats.

Table 4 shows the presence of the neurotransmitters in the gut microbiota. Apart from the abundant presence of amino acids (Phe, Tyr, Trp, and Glu), the concentrations of transmitters varied a lot in the rat fecal samples, including Dopa (1660 ± 253 ng/g), DA (263.8 ± 76.3 ng/g), MLT (1.48 ± 1.88 ng/g), 5-HT (806.5 ± 34.1 ng/g), GABA (11.89 ± 0.51 μg/g), and Ach (8.04 ± 6.96 ng/g). The level of MT, derived from Phe, was 11.19 ± 1.36 ng/g. However, the other metabolites in this pathway, such as adrenaline and norepinephrine etc., were undetectable, which is different from the in vivo results. In terms of the Trp metabolites, the levels of 5-HIAA and 5-HTP (which are derived from 5-HT) in feces were 17.76 ± 2.18 μg/g and 13.82 ± 5.14 ng/g, respectively; KN and KYNA (which are derived from Trp) were 139.3 ± 10.5 ng/g and 1051 ± 116 ng/g, respectively; and the levels of TA, IAA, ILA, and IPA ranged from 1067 ± 111 ng/g to 8833 ± 1531 ng/g. While, if the animals were depressed, then differences in the concentrations of these metabolites were observed, and the results are summarized in Figure 3b,c. The average concentrations of all these amino acids showed a decreasing trend of −36% for Phe, −49% for Tyr (*P* < 0.05), −29% for Trp, and −34% for Glu (*p* < 0.01). The level of Dopa showed a significant increase in the model group, with an average of +105% (*p* < 0.001). Regarding the Trp metabolic pathway, the levels of 5-HT and 5-HIAA were downregulated by −20% (*p* < 0.001) and −30% (*p* < 0.01), respectively, in the depression model. The levels of KN and KYNA decreased as well (63.0 vs. 139.3 ng/g and 668 vs. 1051 ng/g, respectively, *p* < 0.001). In addition, the production of IAA, IPA, and ILA was significantly decreased (−33%, −36%, and −34%, respectively; *p* < 0.01, *p* < 0.05, and *p* < 0.001). These data suggest the variations in the gut microbiota or in the function of the gut under disease. Apart from the above results, the ratio of KYNA/KN levels was elevated in the depression model (in Figure 3d). This result was consistent with the hypothesis that the KYNA/KN ratio is a potential marker for the degree of depression [42].

Several previous studies have shown that abnormal behaviors are not only manifested in changes in brain neurotransmitter levels but are also accompanied by intestinal dysfunction and imbalance of the gut flora [43,44,45]. This phenomenon could be partly explained by the “gut–brain axis” theory, which proposes that a series of signaling pathways mediate the communication between the intestinal bacteria and brain. Interestingly, under depression, changes in the metabolic pathways in the gut microbiota seemed to be concentrated on Trp. This phenomenon is similar to the well-known observation that the levels of 5-HT, 5-HIAA, or their corresponding receptors in brain are disordered under depression [46,47]. Meanwhile, the downregulation of “Trp metabolic” bacteria, namely, *Clostridium* spp., *Bifidobacterium* spp., *Escherichia* spp., *Ruminococcus* spp., and *Lactobacilli* spp., etc., have been reported by several independent investigations in depression patients [48,49,50]. However, whether the change in the composition of the gut microbiota is the reason for, or the result of, the development of mental illness requires further and deeper exploration.

### 2.4. Screening of Postlisting Drugs In Vitro

**Figure 4:**
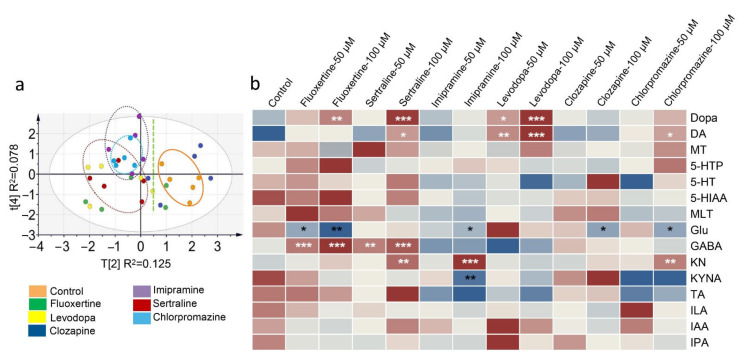
Differences in the levels of transmitters and metabolites in the gut microbiota after first-line drug treatment. (**a**) Principal component analysis score plot showing the diversity of the compound levels among the control group and drug-treated groups (100 μM). (**b**) Heatmap of the compound level. Significant differences of all the drug-treated groups were analyzed by comparison with the control group, and “*” labeled in white means an increase in level, while “*” in black means a decrease in level. * *p* < 0.05, ** *p* < 0.01, *** *p* < 0.001. Data in b were analyzed by two-tailed Student’s *t* test.

Several guideline suggested first-line drugs that treat NVS diseases have been screened for their regulatory potential of gut microbiota [51]. Sertraline and fluoxetine are selective serotonin reuptake inhibitors (SSRIs), and imipramine is a nerve terminal reuptake inhibitor; the indications of these three drugs are depression in adults. Levodopa is a typical anti-Parkinson’s disease drug, using direct transmitter supplementation. Clozapine is a nonclassical neuroblocker for schizophrenia, and chlorpromazine is an antagonist of central dopamine receptors in alleviating schizophrenia symptoms. In Figure 4a, all the samples are within the 95% confidence level, and among them, sertraline, imipramine, and chlorpromazine exhibited preferable regulatory potential for neurotransmitters and metabolites. The transmitters or metabolites that showed variations in level are shown in the heatmap (Figure 4b). Both groups of the SSRIs showed a significant increase in the levels of GABA (*p* < 0.001) and Dopa (*p* < 0.01 and *p* < 0.001), an increase in DA plus KN, and a decrease in Glu in the gut microbiota. High-dose imipramine (100 μM) showed an obvious downregulation of the KYNA/KN ratio (*p* < 0.001). Levodopa did not have an obvious effect on the metabolites in the Trp or Glu pathways, and similar results were observed in the clozapine groups. In addition, the high dose of chlorpromazine induced the production of DA (*p* < 0.05) and KN (*p* < 0.01), and simultaneously decreased the Glu level (*p* < 0.05) in the gut microbiota.

## 3. Materials and Methods

### 3.1. Reagents and Materials

Melatonin (MLT), acetylcholine (Ach), and gamma-aminobutyric acid (GABA) were purchased from Solarbio Scientific Ltd. (Beijing, China). l-dopa (Dopa), dopamine (DA), hydroxytryptophan (5-HTP), 5-hydroxytryptamine (5-HT), and 4-hydroxybenzylamine (internal standard, IS) were obtained from J&K Scientific Ltd. (Beijing, China). Phenylalanine (Phe), tyrosine (Tyr), glutamic acid (Glu), and tryptophan (Trp) were obtained from the National Institutes for Food and Drug Control (Beijing, China). 3-Methoxytyramine (MT), 5-hydroxyindole-3-acetic acid (5-HIAA), kynurenine (KN), kynurenic acid (KYNA), and tryptamine (TA) were obtained from Shanghai Haohong Biological Medicine Technology Co., LTD (Shanghai, China). Indole-3-lactic acid (ILA), indole-3-acetic acid (IAA), and indolyl-3-propionic acid (IPA) were purchased from Shanghai Macklin Biochemical Co., Ltd. (Shanghai, China). The purities of all the reference standards were greater than 98%. Formic acid (100%) was purchased from Merck (Darmstadt, Germany). Acetonitrile and methanol were obtained from Fisher Scientific (HPLC grade, Fair Lawn, NJ, USA). Deionized distilled water was purchased from Hangzhou Wahaha Group Co. Ltd. (Hangzhou, China). All the other chemical reagents were obtained from Sinopharm Chemical Reagent Co., Ltd. (Beijing, China) at the purest degree available. The structural formulas of the nineteen monoamine compounds and IS are shown in Figure 1.

### 3.2. Animals

Sprague-Dawley rats (180–220 g, 8 weeks, male) were supplied by Beijing Vital River Laboratory Animal Technology Co., Ltd. (Beijing, China). All the animals had free access to food and water. The temperature was maintained at 22–24 °C with a 12 h light/dark cycle, and the relative humidity was 40–60%. Fresh fecal samples were collected in sterile nitrogen-filled self-sealing bags and kept at −70 °C. The research complied with the Institutional Guidelines and Ethics, and Laboratory Institutional Animal Care and Use Committees of the Chinese Academy of Medical Sciences and Peking Union Medical College (No. 00003053).

### 3.3. Instruments and LC-MS/MS Conditions

Liquid chromatography with tandem mass spectrometry LC-MS/MS 8060 (Shimadzu Corporation, Kyoto, Japan) with an electrospray ionization (ESI) source was conducted for analysis. An xSelect HSS PFP column (with fluoro-phenyl bonded) was used for separation (100 mm × 2.0 mm × 1.8 μm, Waters, Milford, USA). The flow rate was 0.3 mL/min, and the column temperature was maintained at 35 °C. The mobile phase was formic acid:water (0.2:100, *v/v*) as phase A, and methanol:acetonitrile (1:1) with 0.2% formic acid as phase B. The binary gradient elution conditions were as follows (A:B): 0.01 min, 98:2; 3.50 min, 98:2; 4.00 min, 10:90; 4.50 min, 2:98; 6.00 min, 2:98; 6.50 min, 98:2; 8.50 min, and controller stop. Multiple reaction monitoring (MRM) in the positive mode was performed for detection, and the optimized MRM parameters for each compound are shown in Table 1. The mass condition parameters were set as follows: nebulizer gas, 2.7 L/min; drying gas, 10.0 L/min; heating gas, 10.0 L/min; interface temperature, 300 °C; collision-induced dissociation (CID) gas, 230 kPa; DL temperature, 250 °C; heat block temperature, 400 °C; and interface voltage, −4.5 kV. The autosampler was maintained at 4 °C.

### 3.4. Stock Solutions, Calibration Curve Standards

The neurotransmitter standards, including Dopa, DA, MT, Trp, 5-HTP, 5-HT, 5-HIAA, MLT, KN, TA, ILA, IAA, IPA, GABA, and Ach, were dissolved in a 0.2% formic acid–water solution to obtain a concentration of 1.00 mg/mL. Phe, Tyr, Glu, and KYNA were dissolved in a 0.2% formic acid–methanol solution to a final concentration of 1.00 mg/mL. The stock solution was prepared by thoroughly mixing the standards according to the proportion, with Phe, Tyr, Trp, and Glu 50,000 ng/mL; 5-HIAA, ILA, IAA, IPA, and GABA 2000 ng/mL; Dopa, DA, 5-HTP, 5-HT, KN, TA, and Ach 500 ng/mL; MT, 200 ng/mL; KYNA, 100 ng/mL; and MLT, 10 ng/mL. Eight calibration curve standards (working solutions) were prepared by series dilution, and the final concentrations of Dopa in the mixed standards were as follows: 500 ng/mL, 200 ng/mL, 100 ng/mL, 50 ng/mL, 10 ng/mL, 5 ng/mL, 2 ng/mL, and 0.5 ng/mL. The levels of other substances in the working solutions achieved the corresponding values. The concentrations of the QC for each compound, namely, the low-concentration quality control (LQC), middle-concentration quality control (MQC), and high-concentration quality control (HQC), are listed.

### 3.5. Sample Preparation

Fresh fecal samples were prepared by diluting with 5-fold (*w:v* = 1:5) formic acid–water (*v:v* = 0.2:100). After thoroughly mixing by vortexing and centrifuging at 12,000× *g* for 5 min, the diluted fecal sample (50 μL) was immediately added to 150 μL cold acetonitrile (with 2% formic acid and 100 ng/mL 4-hydroxybenzylamine as IS). The resulting solution was centrifuged at 12,000× *g* for 5 min at 4 °C, and the supernatant was collected into another 1.5 mL tube. Then, the solvent of the collected solution was removed under a flow of nitrogen. The residue was redissolved in 50 μL of the initial mobile phase (with 2% formic acid added) and centrifuged at 12,000× *g* for 5 min at 4 °C. Five microliters of the supernatant was injected for analysis. For QC samples, fecal samples from six rats were first mixed and diluted with a 5-fold (weight:volume = 1:5) of formic acid–water (*v:v* = 0.2:100). Then, 50 μL of the working solutions with different levels of standards were added to an aliquot of the mixed fecal sample (50 μL). Then, 150 μL of cold acetonitrile (with 2% formic acid and 100 ng/mL IS) was added and treated as described above.

### 3.6. Method Validation

#### 3.6.1. Specificity and Carryover

Specificity was evaluated by spiking standards into the fecal matrix, and comparing the chromatograms of the spiked samples with those of the unspiked matrix and standards in solvent. The carryover effect was investigated by injecting the blank solvent after five consecutive injections of HQCs, and a residual response not exceeding 20% of instrumental LLOQ was regarded as acceptable [52].

#### 3.6.2. Linearity

The eight levels of calibration curve standards used for evaluating linearity included Phe, Tyr, Trp and Glu 50,000, 20,000, 10,000, 5000, 1000, 500, 200, and 50 ng/mL; 5-HIAA, ILA, IAA, IPA and GABA 2000, 800, 400, 200, 40, 20, 8, and 2 ng/mL; Dopa, DA, 5-HTP, 5-HT, KN, TA and Ach 500, 200, 100, 50, 10, 5, 2, and 0.5 ng/mL; MT 200, 80, 40, 20, 4, 2, 0.8, and 0.2 ng/mL; KYNA 100, 40, 20, 10, 2, 1, 0.4, and 0.1 ng/mL; and MLT 10, 4, 2, 1, 0.2, 0.1, 0.04, and 0.01 ng/mL. The nonzero calibration curve was built by plotting the peak area ratio of analyte to IS (Y) versus the nominal level (X) [53]. The correlation coefficient (R^2^) was obtained to evaluate linearity, and a weighting factor of 1/X was used for fitting. The linear range was accepted when the relative error of the calibrators was within ±15% by comparison with the theoretical concentrations, except at the lowest limit of quantification (LLOQ, ± 20%) in each run [35,52]. The lowest level of standards on the validated calibration curve was defined as the LLOQ of this method at which an acceptable accuracy (RE) and precision (RSD) could be obtained [54].

#### 3.6.3. Limits of Detection and Quantification (Instrumental)

The instrumental LLOD and LLOQ were assessed according to the guidelines that a signal-to-noise ratio (S/N) of each compound above three was recorded as the LLOD, and S/N above ten was regarded as LLOQ of the instrument [55]. As fecal samples free from the nineteen compounds were not available, solvent standards were used to evaluate the instrumental LLOD and LLOQ.

#### 3.6.4. Accuracy and Precision

Accuracy and precision included two parts: intra-day and inter-day accuracy and precision. Three levels of QC samples (LQCs, MQCs, and HQCs) in five repeats were evaluated on one day and three consecutive days. Accuracy was evaluated by comparing the measured value (*C_1_*) and theoretical value (*C_2_*), and it is expressed as relative error, RE = (*C_1_ − C_2_*)*/C_2_ ×* 100%. Precision is expressed as RSD (%). The accuracy and precision result was accepted when the RE and RSD of QCs were within ±15% by comparison with the theoretical concentrations in each run [56].

#### 3.6.5. Matrix Effects and Extraction Recovery

For endogenous metabolites, the matrix effects and extraction recovery were determined using unspiked samples and standard-spiked samples (LQCs, MQCs, HQCs) in five repeats [52]. The matrix effect was assessed by separately determining the response (peak area) of each compound in unspiked samples (*A*_1_), standard-spiked samples (*A*_2_), and standards free from matrix (*A*_3_) after extraction. The value of the matrix effect was calculated by *A*_2_/(*A*_1_ + *A*_3_) × 100%. The extraction recovery of each analyte was evaluated in five replicates by comparing the peak area of standard-spiked samples before (*A*_4_) and after extraction (*A*_5_), and recovery was calculated by *A*_5_/*A*_4_ × 100%. The spiked samples for *A*_5_ were prepared by QCs. The spiked samples for *A*_4_ were prepared as follows: a diluted fecal sample (50 μL) was added to 150 μL cold acetonitrile (with 2% formic acid and 100 ng/mL IS). After centrifugation and drying, the residue was dissolved in 50 μL of standards at the corresponding levels. Recovery above 60% and deviation in different levels within 15% were regarded as acceptable [57].

#### 3.6.6. Stability

Stability was evaluated using spiked samples (LQCs, MQCs, and HQCs) in triplicate that were placed at 4 °C for 12 h after the addition of cold acetonitrile (2% formic acid) or after preparation (in waiting for the analysis), or maintained at −70 °C for 2 weeks before treatment. Stability was calculated by the ratio of the peak area of each compound after placement to the measured value of the corresponding samples before placement. Data in the range of 85–115% after placement were regarded stable [52,53].

### 3.7. Establishment of the Depression Model

Twelve Sprague-Dawley rats (male, 8 weeks) were randomly separated into two groups to establish a chronic unpredictable stress depression model. Six rats were set as the normal control group (Group 1), and the other six rats were set as the depression model group (Group 2). Animals in group 2 were exposed to two different kinds of unpredictable stresses every day: light/dark reversed, food and water deprivation, ice water swimming, electric shock, behavioral constraints, cold stimulation, clip tail, wet bedding, and slope [58,59]. After continuous modeling for 8 weeks, the sucrose preference test was performed to examine the model after 8 weeks [58,59]. Then, fecal samples were collected from each group for the analysis of the nineteen neurotransmitters and metabolites by LC-MS/MS.

### 3.8. Screening and Evaluation of Postlisting Compounds

Six postlisting drugs, including sertraline hydrochloride (Shanghai Macklin Biochemical Co., Ltd., Shanghai, China), clozapine (Shanghai Yuanye Biological Technology Co., LTD, Shanghai, China), levodopa (J&K Technology Co. LTD, Beijing, China), chlorpromazine hydrochloride (Macklin, Shanghai, China), imipramine hydrochloride (Macklin, Shanghai, China), and fluoxertine hydrochloride (Yuanye, Shanghai, China), were used for drug screening. Five grams of the colon contents from six Sprague-Dawley rats (male, eight weeks) was collected under sterile conditions to isolate intestinal bacteria. After adding 100 mL of sterile anaerobic culture medium (Solarbio Biotechnology Co., LTD, Beijing, China) under a nitrogen atmosphere, the resulting mixture was filtered through gauze to remove the food debris. The intestinal bacterial culture was preincubated for 30 min. Then, 10 μL of standards (in methanol, 5 mM and 10 mM) was added to 990 μL of the intestinal bacterial culture and incubated for 12 h under anaerobic conditions [60]. Finally, the resulting culture was immediately mixed with cold acetonitrile (with 2% formic acid and IS) and treated as described above.

### 3.9. Statistical Analysis

Statistical analysis was performed by GraphPad Prism version 5.72 (GraphPad Software, La Jolla, CA, USA) and SIMCA version 14 (MKS Umetrics AB, Umea, Sweden). Data are expressed as the mean ± standard deviation (SD) and were analyzed by two-tailed Student’s *t* test. A *P* value less than 0.05 was regarded as statistically significant for all the tests. The heatmap was constructed to express the compound levels in different colors, of which blue represents a lower level and red represents a higher level. The drug screening data were first normalized to the sum of total intensity, and then subjected to principal component analysis (PCA). The PCA score plot was built based on the 35 samples (n = 5 in each group, including the control group and six groups with 100 μM drug treatment) and those targeted features measured in the samples. The total variance retained in the four PCs was 0.833 with Q^2^ = 0.436.

## 4. Conclusions

Herein, a novel, simple, and underivatized LC-MS/MS method for the quantification of nineteen monoamine neurotransmitters and metabolites related to the NVS was developed. The method was suitable for intestinal bacterial samples and focused on three pathways, namely, Phe, Trp, and Glu. The compounds were separated on a fluoro-phenyl bonded column, and the linear range for each compound was adjusted according to its level in practical samples. After optimization of the detecting conditions and pretreatment procedures, the compounds exhibited symmetric peak shapes and sufficient sensitivity under a total analysis time of 8.5 min. The method was fully validated with acceptable linearity, accuracy, precision, matrix effect, extraction recovery, and stability. The results of the fecal samples showed that neurotransmitters such as Dopa, DA, 5-HT, GABA, and Ach were present in the gut microbiota. The metabolic pathway of Trp was disordered under depression, with lower levels of 5-HT, 5-HIAA, KN, KYNA, TA, ILA, IAA, IPA, Glu, and Tyr, and a higher ratio of KYNA/KN. In addition, some first-line NVS drugs, such as sertraline, imipramine, and chlorpromazine, showed regulatory potential on these pathways in the gut microbiota as well.

## Figures and Tables

**Table 1:** Optimized multiple reaction monitoring (MRM) condition and mass spectrometer parameters (CE: Collision energy).

Analyte	Formula	Molecular Weight (MW)	MRM (*m/z*)	Q1 CE (Volt)	CE (Volt)	Q3 CE (Volt)
Phenylalanine (Phe)	C_9_H_11_NO_2_	165.2	166.05→120.20	−1	−1	−2
Tyrosine (Tyr)	C_9_H_11_NO_3_	181.2	181.90→136.20	−1	−1	−1
l-dopa (Dopa)	C_9_H_11_NO_4_	197.2	198.05→152.25	−23	−14	−30
Dopamine (DA)	C_8_H_11_NO_2_	153.2	154.10→119.20	−11	−20	−11
3-Methoxytyramine (MT)	C_9_H_13_NO_2_	167.2	168.10→151.20	−17	−12	−14
Tryptophan (Trp)	C_11_H_12_N_2_O_2_	204.2	205.25→188.25	−1	−1	−1
5-Hydroxytryptophan (5-HTP)	C_11_H_12_N_2_O_3_	220.2	220.90→134.20	−11	−25	−25
5-Hydroxytryptamine (5-HT)	C_10_H_12_N_2_O	176.2	177.25→160.20	−18	−11	−30
5-Hydroxyindole-3-acetic acid (5-HIAA)	C_10_H_9_N_2_O_3_	191.2	192.00→146.20	−13	−15	−14
Melatonin (MLT)	C_13_H_16_NO_2_	232.3	233.00→174.10	−12	−15	−16
Kynurenine (KN)	C_9_H_7_NO_5_	208.2	208.90→146.10	−16	−20	−24
Kynurenic acid (KYNA)	C_10_H_7_NO_3_	189.2	190.20→143.90	−10	−19	−26
Tryptamine (TA)	C_10_H_12_N_2_	160.2	161.10→144.20	−30	−11	−13
Indole-3-lactic acid (ILA)	C_11_H_11_NO_3_	205.2	205.90→130.10	−15	−27	−25
Indole-3-acetic acid (IAA)	C_10_H_9_NO_2_	175.2	175.90→130.00	−12	−17	−22
Indolyl-3-propionic acid (IPA)	C_11_H_11_NO_2_	189.2	190.10→130.00	−13	−20	−12
Glutamic acid (Glu)	C_5_H_9_NO_4_	147.1	148.10→84.10	−2	−1	−2
Gamma-aminobutyric acid (GABA)	C_4_H_9_NO_2_	103.1	104.10→87.20	−22	−12	−16
Acetylcholine (Ach)	C_7_H_16_NO_2_	146.2	146.10→60.00	−10	−12	−23
4-Hydroxybenzylamine (IS)	C_7_H_9_NO	123.2	124.30→107.00	−17	−12	−10

**Table 2:** Retention time, and linear range for each compound.

Analyte	Rt (min)	Linear Range (ng/mL)	R^2^	Instrumental Lowest Limit of Detection (LLOD) (ng/mL)	Instrumental Lowest Limit of Quantification (LLOQ) (ng/mL)
Phe	3.31	500–50,000	0.9923	2	5
Tyr	2.18	200–50,000	0.9901	5	10
Dopa	1.69	2–500	0.9961	0.2	0.5
DA	2.14	5–500	0.9982	0.2	0.5
MT	5.25	0.2–200	0.9948	0.08	0.2
Trp	6.35	200–50,000	0.9901	2	5
5-HTP	5.11	0.5–500	0.9980	0.2	0.5
5-HT	5.90	2–500	0.9926	0.1	0.2
5-HIAA	5.96	20–2000	0.9908	0.4	0.8
MLT	6.21	0.01–10	0.9973	0.005	0.01
KN	1.84	2–500	0.9912	0.1	0.5
KYNA	6.11	1–100	0.9941	0.02	0.1
TA	6.56	5–500	0.9918	0.1	0.5
ILA	6.03	20–2000	0.9912	0.05	0.2
IAA	6.09	20–2000	0.9912	0.01	0.05
IPA	6.16	20–2000	0.9966	0.1	0.5
Glu	0.98	500–50,000	0.9987	1	2
GABA	1.09	20–2000	0.9994	0.4	0.8
Ach	2.55	2–500	0.9902	0.1	0.2

**Table 3:** Accuracy, precision, matrix effects, extraction recovery, and stability (RE: Relative error; RSD: Relative standard deviation).

Analyte	Concentration (ng/mL)	Intra-Day (n = 5)		Inter-Day (n = 15)		Matrix Effects	Extraction Recovery	Stability after Treatment (4 ℃, 12 h)	Stability after Added with Acetonitrile (4 ℃, 12 h)	Freeze Stability (−70 ℃, 2 Weeks)
		Accuracy (RE%)	RSD (%)	Accuracy (RE%)	RSD (%)	Mean ± SD (%)	Mean ± SD (%)	Mean ± SD (%)	Mean ± SD (%)	Mean ± SD (%)
Phe	500	−11.58	1.57	−11.27	2.16	112.80 ± 6.95	104.27 ± 3.07	112.61 ± 3.92	97.47 ± 3.82	97.28 ± 6.99
	5000	3.23	4.70	1.76	5.23	103.65 ± 3.72	107.93 ± 3.20	100.61 ± 2.34	92.85 ± 8.35	96.19 ± 2.92
	50,000	−5.84	4.99	−7.33	4.10	84.17 ± 2.84	100.44 ± 8.13	114.73 ± 2.97	96.90 ± 9.27	107.34 ± 5.02
Tyr	200	5.17	6.43	1.45	10.42	82.00 ± 4.05	103.99 ± 5.75	93.07 ± 7.29	90.55 ± 6.44	99.36 ± 4.13
	5000	1.25	5.22	−2.29	6.44	95.95 ± 10.73	109.05 ± 3.45	93.11 ± 3.53	91.16 ± 4.34	85.61 ± 2.54
	50,000	−4.43	6.54	−4.43	9.31	91.81 ± 13.82	95.57 ± 6.54	95.45 ± 2.38	93.52 ± 2.30	93.93 ± 4.57
Dopa	2	0.65	4.60	2.08	6.11	102.52 ± 11.55	98.97 ± 2.41	99.40 ± 10.29	105.37 ± 4.77	99.63 ± 5.78
	50	2.93	2.85	5.37	5.08	104.59 ± 2.52	105.58 ± 6.92	105.42 ± 3.90	91.49 ± 7.46	106.57 ± 7.56
	500	−2.87	5.03	−0.51	9.73	91.30 ± 14.60	90.40 ± 6.86	99.58 ± 3.90	96.94 ± 14.32	87.37 ± 6.15
DA	5	−5.24	4.03	0.00	7.86	87.08 ± 6.63	101.42 ± 2.89	89.49 ± 3.23	102.33 ± 11.46	100.29 ± 6.19
	50	−6.81	2.35	−3.03	6.55	117.71 ± 2.59	104.50 ± 3.75	105.34 ± 2.17	95.78 ± 5.44	96.98 ± 7.29
	500	−0.85	4.69	4.93	6.48	99.98 ± 2.70	98.81 ± 7.06	99.30 ± 7.12	95.89 ± 6.88	87.65 ± 2.08
MT	0.2	0.52	8.91	1.96	8.15	96.35 ± 11.01	104.32 ± 2.90	99.00 ± 7.59	98.73 ± 6.09	97.40 ± 4.75
	20	−2.66	2.28	−0.38	7.38	105.30 ± 4.44	117.21 ± 12.74	109.28 ± 3.23	87.67 ± 2.47	97.33 ± 10.29
	200	−2.77	5.53	2.64	8.07	98.58 ± 7.20	101.69 ± 7.27	103.39 ± 8.57	97.29 ± 7.90	87.58 ± 4.27
Trp	200	−0.51	6.77	−1.08	8.13	113.90 ± 11.90	101.85 ± 2.64	87.13 ± 5.00	100.84 ± 6.03	95.71 ± 9.60
	5000	0.52	5.39	4.11	6.14	108.70 ± 3.72	97.31 ± 9.13	86.55 ± 3.60	97.37 ± 5.63	85.40 ± 2.58
	50,000	−2.26	8.88	−7.67	6.87	102.22 ± 5.95	97.74 ± 8.88	98.28 ± 6.45	100.48 ± 3.76	103.69 ± 6.40
5-HTP	0.5	4.00	4.87	1.52	6.27	111.75 ± 8.99	105.38 ± 1.48	92.07 ± 0.77	96.40 ± 10.88	98.20 ± 9.55
	50	0.62	3.32	−0.48	5.41	98.58 ± 3.29	102.07 ± 7.32	101.27 ± 3.65	92.41 ± 8.78	89.47 ± 0.77
	500	5.94	6.25	1.08	8.55	97.38 ± 3.07	101.20 ± 6.91	101.16 ± 3.38	93.45 ± 7.81	97.54 ± 8.10
5-HT	2	0.25	7.66	−0.77	5.71	99.30 ± 4.78	101.59 ± 3.47	103.88 ± 10.32	100.90 ± 7.96	101.08 ± 7.07
	50	−6.16	3.87	−0.95	7.46	90.88 ± 3.93	95.61 ± 5.43	93.84 ± 5.14	93.12 ± 11.14	96.01 ± 10.78
	500	−5.01	10.15	3.64	10.31	112.20 ± 2.32	108.20 ± 7.77	92.51 ± 2.15	95.28 ± 14.95	86.16 ± 5.13
5-HIAA	20	1.07	6.53	−0.35	7.68	71.91 ± 5.59	105.93 ± 2.78	94.18 ± 1.46	99.95 ± 2.78	98.50 ± 1.94
	200	−3.34	1.70	−3.72	6.35	82.20 ± 11.50	100.51 ± 1.49	93.31 ± 0.96	97.08 ± 0.49	96.84 ± 0.32
	2000	−0.03	5.34	2.19	5.91	71.06 ± 2.89	103.75 ± 3.69	87.36 ± 4.79	104.24 ± 5.55	93.36 ± 4.12
MLT	0.01	4.32	7.90	1.40	9.66	83.57 ± 8.83	98.22 ± 10.39	106.33 ± 10.56	94.60 ± 6.36	102.27 ± 15.61
	1	−2.23	5.81	−1.75	5.82	74.20 ± 3.07	98.97 ± 6.41	102.88 ± 10.45	99.43 ± 4.81	99.72 ± 9.47
	10	−4.10	7.14	1.06	8.21	68.24 ± 4.50	98.76 ± 8.47	96.16 ± 8.44	87.07 ± 5.59	86.38 ± 3.97
KN	2	7.46	3.69	−0.66	8.21	105.66 ± 3.48	84.81 ± 8.05	97.78 ± 7.71	94.41 ± 7.36	108.12 ± 4.06
	50	3.41	4.44	1.58	6.24	98.29 ± 9.37	94.35 ± 5.43	101.82 ± 4.29	93.13 ± 3.80	108.37 ± 5.38
	500	2.36	5.00	6.69	5.00	99.18 ± 9.53	96.09 ± 5.54	91.38 ± 3.66	89.70 ± 0.82	104.05 ± 6.40
KYNA	1	−0.50	9.58	−1.29	7.68	94.82 ± 2.89	96.20 ± 7.79	95.38 ± 2.49	103.28 ± 5.31	107.99 ± 6.10
	10	−2.99	8.83	0.99	8.29	86.52 ± 3.20	95.36 ± 2.28	102.46 ± 2.75	111.28 ± 7.19	111.21 ± 5.71
	100	9.71	5.53	2.27	9.57	90.52 ± 13.77	98.81 ± 6.38	94.82 ± 1.69	104.38 ± 1.93	104.46 ± 2.14
TA	5	7.08	9.72	2.13	9.39	92.64 ± 0.48	107.47 ± 9.48	94.00 ± 9.59	100.49 ± 2.92	107.69 ± 9.97
	50	11.49	1.56	1.75	10.18	114.41 ± 6.28	105.46 ± 5.45	90.61 ± 1.73	111.13 ± 3.85	97.04 ± 11.77
	500	5.05	7.99	5.64	6.19	111.39 ± 11.04	104.36 ± 2.68	92.10 ± 7.09	99.38 ± 5.26	94.59 ± 1.92
ILA	20	5.62	4.82	2.49	8.40	71.28 ± 5.46	102.67 ± 5.35	89.59 ± 6.35	102.96 ± 5.99	103.52 ± 6.03
	200	2.32	7.14	2.89	8.02	75.23 ± 13.26	100.13 ± 5.86	102.75 ± 2.92	103.98 ± 9.56	106.87 ± 6.41
	2000	−2.51	7.08	2.15	7.24	75.45 ± 6.69	100.21 ± 4.46	91.26 ± 1.57	85.19 ± 0.42	102.68 ± 0.37
IAA	20	8.85	6.81	4.20	9.17	102.01 ± 14.26	99.97 ± 5.70	96.60 ± 1.01	101.00 ± 6.20	95.08 ± 9.72
	200	2.70	6.59	5.36	8.57	114.53 ± 3.20	102.43 ± 3.40	96.04 ± 1.47	104.02 ± 2.12	91.42 ± 5.67
	2000	3.49	7.09	5.37	7.14	109.47 ± 6.81	101.81 ± 6.25	94.66 ± 4.56	92.29 ± 2.70	96.70 ± 4.99
IPA	20	4.56	7.80	0.36	8.46	75.49 ± 3.41	114.75 ± 7.92	113.84 ± 2.70	105.50 ± 3.61	114.78 ± 2.58
	200	0.46	7.39	−0.38	8.01	78.12 ± 10.83	114.36 ± 5.16	95.96 ± 2.05	110.57 ± 4.60	101.65 ± 11.79
	2000	−5.83	6.39	−0.79	6.51	77.22 ± 1.72	105.51 ± 5.49	97.83 ± 0.86	92.98 ± 1.91	105.37 ± 3.66
Glu	500	−7.35	7.82	−2.31	9.88	80.04 ± 11.12	97.30 ± 1.71	89.09 ± 3.05	96.77 ± 6.49	91.24 ± 2.77
	5000	1.59	5.58	5.10	7.21	75.98 ± 1.84	97.18 ± 11.02	98.30 ± 4.17	101.67 ± 2.47	100.82 ± 4.50
	50,000	−4.26	7.06	−2.67	9.84	72.91 ± 1.39	104.99 ± 13.04	106.21 ± 4.08	94.91 ± 8.60	96.74 ± 7.96
GABA	20	5.61	10.09	3.31	9.05	103.75 ± 3.85	94.14 ± 7.88	92.98 ± 2.73	107.19 ± 10.78	97.63 ± 10.89
	200	0.87	6.06	−2.21	7.54	99.92 ± 3.10	100.30 ± 5.14	92.03 ± 1.59	98.99 ± 2.92	87.76 ± 2.92
	2000	5.10	8.91	4.00	7.37	102.40 ± 9.73	83.14 ± 4.75	91.61 ± 1.22	103.20 ± 4.46	93.34 ± 6.30
Ach	2	0.25	7.09	1.78	8.45	105.66 ± 10.21	106.85 ± 6.64	104.18 ± 9.45	113.23 ± 7.70	103.03 ± 5.85
	50	−0.47	8.03	3.26	7.53	103.02 ± 3.77	100.55 ± 4.97	109.91 ± 1.68	105.11 ± 9.55	102.20 ± 6.10
	500	−3.87	6.55	−4.03	5.86	95.46 ± 3.66	102.77 ± 8.59	97.69 ± 6.59	94.41 ± 12.30	86.83 ± 4.35

**Table 4:** The level of transmitters and metabolites in the rat feces of normal control and depression model rats.

	Control	Model
Phe	13.85 ± 4.57 μg/g	8.92 ± 8.07 μg/g
Tyr	13.07 ± 3.50 μg/g	6.60 ± 5.47μg/g
Dopa	1660 ± 253 ng/g	3409 ± 266 ng/g
DA	263.8 ± 76.3 ng/g	216.8 ± 48.0 ng/g
MT	11.19 ± 1.36 ng/g	12.88 ± 1.65 ng/g
Trp	1409 ± 277 ng/g	1000 ± 756 ng/g
5-HTP	13.82 ± 5.14 ng/g	11.79 ± 2.44 ng/g
5-HT	806.5 ± 34.1 ng/g	642.1 ± 64.4 ng/g
5-HIAA	17.76 ± 2.18 μg/g	12.47 ± 1.74 μg/g
MLT	1.48 ± 1.88 ng/g	1.25 ± 1.15 ng/g
KN	139.3 ± 10.5 ng/g	63.0 ± 4.9 ng/g
KYNA	1051 ± 116 ng/g	668 ± 75 ng/g
TA	2846 ± 195 ng/g	2415 ± 142 ng/g
IAA	8833 ± 1531 ng/g	5934 ± 996 ng/g
IPA	1572 ± 501 ng/g	1008 ± 138 ng/g
ILA	1067 ± 111 ng/g	704 ± 88 ng/g
Glu	583.7 ± 70.9 μg/g	384.4 ± 119.6 μg/g
GABA	11.89 ± 0.51 μg/g	12.81 ± 1.49 μg/g
Ach	8.04 ± 6.96 ng/g	5.85 ± 4.73 ng/g

## Data Availability

The data in this study are available in this article.

## Sample Availability

The standards of the nineteen monoamines are available from the authors.

## Abbreviations

Ach, Acetylcholine; CID, Collision induced dissociation; CE, Collision energy; DA, Dopamine; Dopa, l-dopa; ESI, Electrospray ionization; GABA, Gamma-aminobutyric acid; Glu, Glutamic acid; 5-HIAA, 5-5-Hydroxyindole-3-acetic acid; 5-HT, 5-Hydroxytryptamine; 5-HTP, Hydroxytryptophan; HQC, High-concentration quality control; IAA, Indole-3-acetic acid; ILA, Indole-3-lactic acid; IPA, Indolyl-3-propionic acid; IS, Internal standard; KN, Kynurenine; KYNA, Kynurenic acid; LC-MS/MS, Liquid chromatography–tandem mass spectrometry; LLOD, Lowest limit of detection; LLOQ, Lowest limit of quantification; LQC, Low-concentration quality control; MLT, Melatonine; MQC, Middle-concentration quality control; MRM, Multiple reaction monitoring; MT, 3-Methoxytyramine; MW: Molecular weight; NVS, Nervous system; PCA, Principal component analysis; Phe, Phenylalanine; QC, Quality control; RE, Relative error; RSD, Relative standard derivation; Rt, Retention time; SD, Standard deviation; S/N, Noise ratio; SSRI: Selective serotonin reuptake inhibitors; TA, Tryptamine; Trp, Tryptophan; Tyr, Tyrosine.
